# Structural colour in red seaweeds is more common and diverse than has been presumed

**DOI:** 10.1098/rsif.2025.0342

**Published:** 2025-09-03

**Authors:** Margot Arnould-Pétré, Silvia Vignolini, Juliet Brodie

**Affiliations:** ^1^ Research, Natural History Museum, London, UK; ^2^ Institut des Substances et Organismes de la Mer (ISOMer), UR 2160, Nantes Université, Nantes, France; ^3^ Sustainable and Bio-inspired Materials, Max Planck Institute of Colloids and Interfaces, Potsdam, Germany; ^4^ Department of Chemistry, University of Cambridge, Cambridge, UK

**Keywords:** rhodophytes, marine macroalgae, nanostructured organelles, iridescent bodies, multilayers, structurally coloured macroalgae, structural colour mechanisms, phylogenetics, distribution of structural colour, iridescent seaweeds

## Abstract

The brightest colorations observed in nature are the result of structural colour, a physical phenomenon relying not on pigments but on the interactions of light with nanostructured materials. Research on structural colour in seaweeds has been growing and hints that the phenomenon is considerably more widespread in these organisms than previously understood. In this review, we combine information from published literature, herbarium specimens and our own observations to clearly outline and reframe the current state of knowledge on the phenomenon in red seaweeds (Rhodophyta). We describe structural colour and the structures responsible for it in rhodophytes, identifying clear categories and their variations. Through an overview of the phylogenetic, geographic and ecological distribution of the phenomenon, we confirm that it is more widespread and diverse than had been indicated by casual recording. We finally discuss hypotheses on the biological significance of structural colour for red seaweeds. Our investigation emphasizes the need for more extensive research in order to fully assess the evolutionary mechanisms at play, the development of the nanostructures and their relation to environmental conditions. This review provides a framework for understanding and classifying structural colour in red algae to encourage a more comprehensive reporting of the phenomenon.

## 1. Introduction

Structural coloration, in contrast to colours obtained by pigmentation, arises from the interaction of light with nanostructured materials. In the natural world, an incredible variety of structures, mechanisms and materials have been exploited to achieve nanostructured tissues responsible for the most spectacular optical appearances of living organisms from land to sea.

In recent years, several studies have described this phenomenon in a wide variety of groups of organisms: from insects to plants, to birds and mammals but also in fish and several crustaceans [1–17]. Research into the biological significance of the phenomenon has established that structural colour serves different purposes in those organisms, and other studies have found potential technological applications [1,5]. However, structural colour in other groups, including seaweeds (e.g. [18–22]), fungi [23] and bacteria [24,25] has until recently largely been unexplored.

Seaweeds play a critical role in ecosystems globally, by forming habitats supporting a high diversity of organisms [26], representing a source of oxygen and food for animals including humans [27], and contributing to carbon fixation [28]. As they also have been showcased to have numerous potential applications in sustainable development [29], understanding better their structural colour might be key to mitigating the effects of global change on ecosystems and resources.

Seaweeds represent a large and diverse group of photoautotrophic organisms with over 12 200 species described to date [30,31], which is thought to be approximately half of the existing number of species in the world. While published records show phycologists have been noticing the iridescent colours of some seaweeds for at least 140 years [32–42], and a few authors have previously compiled information on ‘iridescence’ (a typical manifestation of structural colour) in seaweeds in the first half of the twentieth century (e.g. [34,40]), it is only in the last decade that there has been renewed interest in the subject [18–22]. These studies have explored the topic for two well-known structurally coloured seaweeds, the red (Rhodophyta) seaweed *Chondrus crispus* [18,22] and the brown (Phaeophyceae) seaweed *Ericaria selaginoides* [20,21], shedding some light on the physics involved and the potential functions in the biology of these species. Despite these first advances, there is still very little scientific understanding of structural colour in seaweeds, and neither the mechanisms by which it has evolved nor the biological functions have been fully established [18,34,38,40,43]. A review of structural colour with a focus on the brown seaweeds has been published by Chandler *et al.* [19]. In the review we present here, we focus on the phenomenon in the red seaweeds (rhodophytes), which have a very different evolutionary history from the browns.

Specifically, we describe the optical appearance of the phenomenon in these organisms and discuss the different types of structural colour mechanisms found in the group, expand on their phylogenetic distribution, provide an overview of the geographic and ecological distribution of the phenomenon based on literature and herbarium data, and finally discuss its functional purpose.

## 2. Optical appearance of red seaweeds

**Figure 1.**
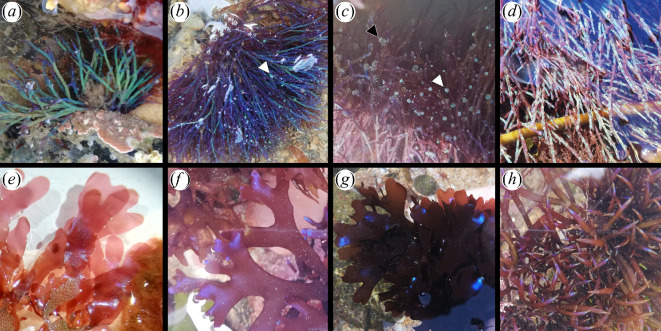
Macroscopic field images of rhodophytes displaying structural coloration. (*a*–*c*) *Chondria coerulescens*. (*a*) Young *C. coerulescens* with green and turquoise structural colour. (*b*) *C. coerulescens* with purple structural colour and turquoise cystocarps (white arrowheads). (*c*) *C. coerulescens* with cystocarps (white arrowheads) and spermatangial plates (black arrowheads). (*d*) *C. scintillans* with turquoise spots of structural colour. (*e*) *Erythroglossum laciniatum* with sparkly blue structural colour on the frond. (*f*,*g*): *Chondrus crispus* with metallic-looking blue structural colour on the apices. (*h*) *Chondracanthus acicularis* with rainbow-coloured apices. Credit: M. Arnould-Pétré

Red seaweeds are classified in the Rhodophyta and are primarily red in colour. This is due to pigments called phycoerythrin and phycocyanin, which are combined with other pigments of the alga (including chlorophyll a, beta-carotene, xanthophylls). The colour produced by pigments depends strictly on the chemical composition of the pigment itself. Each pigment molecule absorbs a specific subset of wavelengths of the incoming light and scatters the wavelengths that are not absorbed, determining the coloration. The red macroscopic appearance of these seaweeds is, therefore, dominated by the presence of pigments, which absorb the green and blue parts of the spectrum, which is why the rhodophytes appear red. In the presence of structural colour, the optical appearance can dramatically change. Superimposed on the red coloration of the seaweed, part of the tissues can display a strong vivid coloration, ranging from purple to navy to light blue, turquoise and green (figure 1).

A simplified but rigorous physical description of structural colour and how it can be measured can be found in other reviews [13]. In summary, structural colour is defined as a physical phenomenon, as it is not only the chemical characteristic of the material which is at play. In principle, every transparent material structured at the length scale of a few hundreds of nanometres can provide structural colours. The colour depends on the nano-scale architecture and the refractive index of the materials composing that architecture. As a consequence, structural coloration, in contrast to pigment, cannot bleach in time and can be destroyed or damaged only if the nanostructure of the material is damaged or modified. For example, structural coloration in herbarium material can be lost if the structure is irreversibly damaged as the tissue becomes dehydrated [44]. In some cases, especially when specimens’ structural colours are caused by extra-cellular nanostructures (cf. §3), the coloration can become visible again if the tissues are rehydrated: for example, structural colour is recovered in the seaweeds *Iridaea flaccida* and *I. cordata* when rehydrated after an extended period of storage dried and frozen [38]. In contrast, pigmented colour cannot be recovered once bleached.

## 3. Types of structures and mechanisms of structural colour in rhodophytes

Various types of mechanisms occur in the production of structural colour in a plethora of organisms in nature [4,6,13,16,45,46]. Each type differs from the others by its complexity, dimensions and physical organization [19]. In red seaweeds, the structural colour has been well described for *Chondrus crispus* [18,47] and in species of *Chondria* [37], *Gastroclonium* [36] and *Iridaea* [38].

**Figure 2.**
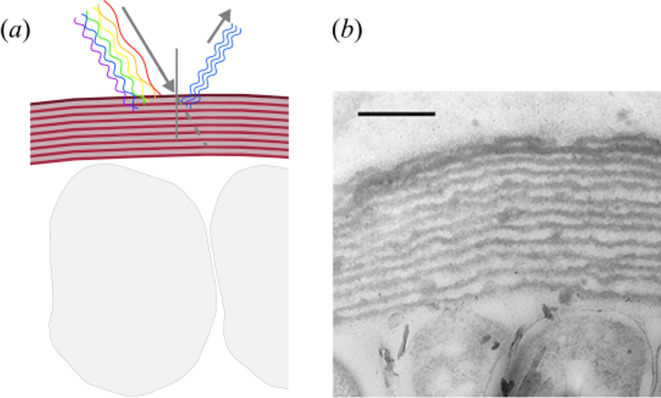
Schematic representations and electron micrograph of an extra-cellular multilayer structure. (*a*) Schematic representation of a multilayer structure above two cells, illustrating the light-scattering mechanism. (*b*) Electron microscopy micrograph of the cuticular multilayer at the tip of the *Chondrus crispus* frond (scale bar: 500 nm), adapted from [18] (under licence CC BY 4.0).

**Figure 3.**
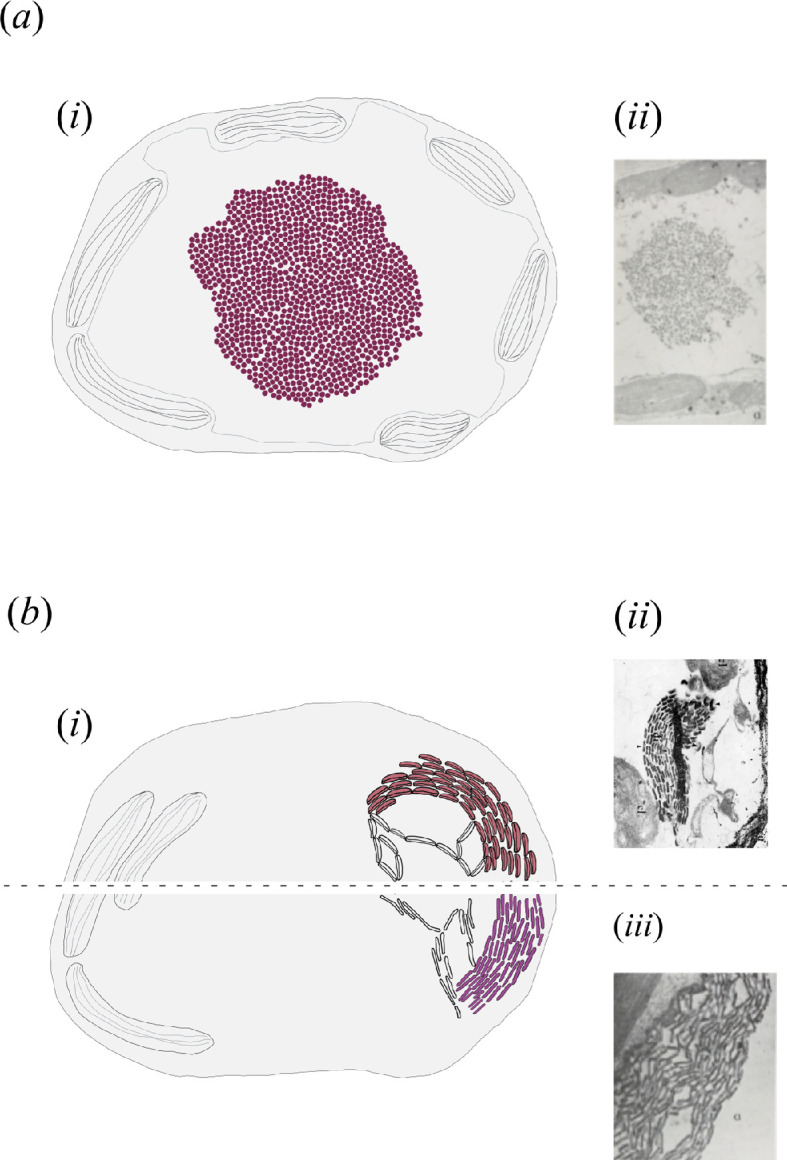
Schematic representations and electron microscopy micrographs of intra-cellular nanostructured organelles. (*a*) Sphere-shaped type: (*i*) schematic drawing of a cluster of spherical nanostructured organelles (coloured) inside a cell. Striped ovals: chloroplasts. (ii) Electron microscopy micrograph of a cluster of nanostructured organelles in *Chondria coerulescens*. Magnification ×10 750. Adapted from [37]. (*b*) Rod- and disc-shaped type: (*i*) schematic drawing of a screen of disc-shaped (upper half) or rod-shaped (lower half) nanostructured organelles inside a cell. Striped ovals: chloroplasts. (ii) Electron microscopy micrograph of nanostructured organelles in *Chylocladia kaliformis* (now *C. verticillata*). Magnification ×7630. Image owned by the Botanical Garden of Naples, University of Naples ‘Federico II’, and adapted from [48], copyright: University of Naples ‘Federico II’. (iii) Electron microscopy micrograph of nanostructured organelles in *Gastroclonium clavatum*. Magnification ×25 740. Adapted from [36].

We distinguish two main categories of structures producing structural colour that have so far been documented in red seaweeds: extra-cellular structures and intra-cellular structures (figures 2 and 3). Extra-cellular structures are found in the form of ‘multilayers’, which consist of a layering of the cell wall where low- and high-density layers with distinct refractive indices are alternated periodically. In contrast, the intra-cellular structures are membrane-isolated organelles containing a more or less periodic organization of sphere-like or rod-like particles. While literature on the physico-chemical properties of the structures in red seaweeds is still too limited to enable an exhaustive description, we do outline some of the overall characteristics within each category, with the aim of facilitating further development of the description of these structures as research on the subject grows. For each of the two categories of structures, we give here examples of common species, the localization of the structure in the seaweed’s anatomy, a description of what is known of the structure’s composition and its mechanism creating structural colour, as well as an overview of the main literature that has focused on investigating it. We also make comparisons with brown seaweeds to explain some of the structures and functions in the reds, but note that they are in a different evolutionary lineage from the browns, which diverged through secondary endosymbiosis about 1300 million years after the reds [49]. The structures involved in the creation of structural colours are therefore unlikely to be homologous.

### 3.1. Extra-cellular structures

Some of the most well-known structurally coloured red seaweeds are found in the order Gigartinales, including in species of the genera *Iridaea* and *Mazzaella* which are widespread in the East Pacific and Southern Ocean, as well as in *Chondrus crispus* which is common on North Atlantic coasts. The visible blue iridescence on the surface of these seaweeds is due to a periodic structural layering of the cuticle in the upper 2 μm of the thalli of the seaweed [38], which creates a vivid metallic-looking structural coloration (figure 1*f,g*).

We call this structure ‘multilayer’ (figure 2). It is composed of periodic alternating layers (with e.g. a thickness of 35−65 and 75−250 nm in *Iridaea* [38]) of two different compositions (therefore different refractive indices). This arrangement causes the light to be scattered back at each layer and to constructively interfere for a specific wavelength, thus producing a strong reflection at that particular wavelength [3]. Pigments may also interfere with the resulting colour in different ways: the structural colour caused by the multilayer may be superimposed on the pigmentation of the tissue, or the pigments may cause a change in the refractive index of the material itself [50]. The physical properties (e.g. thickness, refractive index) of these layers might be very different depending on the species. While the value of the thickness can be estimated from transmission electron microscopy (TEM) cross-sectional imaging, the value of the refractive index can only be estimated indirectly by computing the optical response of the multilayer structure, as it has been done for *C. crispus* in Chandler *et al.* [18]. While this is a general problem in the field of structural colour in nature, future studies providing a better understanding of the local chemical composition of the cell wall might provide more precise values. Multilayered structures were first described in the 1970s for *Iridaea cordata*, *Mazzaella flaccida* and *M. splendens* [38,51]. More recently, the well-known characteristic blue colour observable on the tips of the gametophyte phase in *C. crispus* has been demonstrated to be a structural colour restricted to the apices of the developing fronds. This is where layers are found in higher quantities and stacking is more regularly ordered than in the rest of the thalli, which has no structural colour [18]. It is hypothesized that structural colour in this species might serve as a photoprotective mechanism [22] (cf. §5).

### 3.2. Intra-cellular structures

Other structurally coloured seaweeds well-known by phycologists are found in the order Ceramiales, including notably species of the genus *Chondria* (currently considered widespread around the world, although some taxonomic re-evaluation is needed to verify the currently accepted distribution [52,53]). The bright blue or purple iridescence visible on the branches of these seaweeds (figure 1*a–d*) is due to structures situated inside their cortical cells. While some studies have assumed these structures to be the same as in the brown and the red seaweeds [54], several other studies have shown that this was not the case [55].

Let us first introduce a new term here: ‘nanostructured organelles’. Structural colour created by intra-cellular structures has been reported multiple times for red seaweeds. However, the literature fails to be consistent in the nomenclature used when reporting them. They have often been referred to as ‘iridescent bodies’, which was historically used to describe elements responsible for the blue and purple structural colour in the tegument of some fish and cephalopods [56]. The expression was applied to red seaweeds together with a number of variations, such as ‘iridescent apparatus’, ‘refringent bodies’, ‘iridescent elements’, ‘corpuscles’, ‘light-reflecting bodies’ and also ‘osmophilic granulations’ [35–37,40,43,54,57]. It is important to note also that the use of the term ‘iridescent bodies’ is not strictly correct: by definition, iridescence simply refers to the characteristic of the colour to be angular-dependent, which is not always the case here [19]. While structural coloration can be strongly iridescent, disorder in a perfectly correlated structure can lead to structural colours that are not iridescent and appear uniform regardless of the direction of observation. The interested reader can find more information and references about this phenomenon in the section about structural colour in nature in the review by Vynck *et al.* [58].

Considering the lack of consensus on the nomenclature and the misleading meaning of the terms formerly used for the descriptions of these intra-cellular structures until now, we propose a term which reflects their nature in the rhodophytes: ‘nanostructured organelles’. As a matter of fact, these structures, which fulfil a role in the cell, are defined by their nanoscopic architecture.

In the rhodophytes, the nanostructured organelles are similar across species but not necessarily identical, with subtle variations in the shape, chemical composition and behaviour. Two main types of such structures have been described (figure 3). In the first type (figure 3*a*), sphere-shaped organelles less than 240 nm in diameter are packed together and form a globular cluster, described as being membrane-less, inside the vacuole of the cell [37,40,59]. According to Feldmann-Mazoyer and colleagues [35,37], who described them in *C. scintillans* and *C. coerulescens*, these organelles are themselves formed of granules *ca* 20 nm in diameter. This type of organelle seems restricted to species from the Ceramiales and is always described as an intra-vacuolar element [34,37,40]. In the second type (figure 3*b*), elongated organelles from 100 to 350 nm long and 65 nm thick are assembled together in strings, which are packed in regular parallel rows forming a screen on one side of the cell. This second type of organelle is described as rod-shaped in some species (e.g. *Gastroclonium clavatum* [36]) or disc-shaped in others (e.g. *Chylocladia verticillata* [43]). This type of organelle seems to be found in species from the Rhodymeniales and is found in the cell vacuole in most cases, but sometimes in the cytoplasm (in *G. clavatum* [36]).

The chemical composition of these organelles is unclear. In some species in the Ceramiales, the spherical organelles are sometimes reported as proteinaceous (*Callithamnion granulatum*), sometimes phenolic (*Chondria coerulescens*, *C. scintillans,* and some *Laurencia* species) [34,35,37,40], as is the case in brown seaweeds where they have been studied (e.g. *Cystoseira stricta* (now *Ericaria amentacea*)) [60]. The hypothesis of a lipidic composition is also mentioned [57]. The rod- or disc-shaped organelles in the Rhodymeniales species *G. clavatum* or *Chylocladia verticillata* are not thought to be of the same chemical composition as the Ceramiales [34,40] (species given in the reference as *C. clavata* and *C. kaliformis,* respectively). Again, as in the case of multilayered structures, future studies with more modern tools and techniques could provide more information on the composition.

The role of those nanostructured organelles in the appearance of structural colour was first mentioned by Berthold [32], who recorded them in *Chylocladia* in the late nineteenth century. Studies by Feldmann-Mazoyer & Heim [36,37] and Talarico [43] reported the phenomenon without studying the quantitative optical response of the tissue, simply speculating that the difference in refractive index between the organelles and the surrounding medium inside the vacuole was the cause of the appearance of structural colour. A more extensive optical and anatomical characterization has been performed in the case of the brown seaweed *Cystoseira tamariscifolia* (now *Ericaria selaginoides*) [20], where the structural coloration was directly correlated with the organization in a three-dimensional periodic arrangement of such nanostructured organelles, such as those found in opals. Analogous to the one-dimensional multilayer structure, such periodic arrangement allows a selective reflection of a specific coloration to be achieved due to the interference between scattered light at the interface between the organelles and the surrounding medium [5]. It is important to reiterate that brown and red seaweeds have very different evolutionary histories, and it is unlikely that the structures have resulted from the same evolutionary processes. However, we may find, in the red seaweeds described here, an optical mechanism similar to the one described in *E. selaginoides*. The structure with rod-shaped or disc-shaped organelles assembled in a periodic manner may create structural colour by forming a kind of intra-cellular multilayer [36,40,43] when the refractive index of these organelles assembled in strings is significantly different from that of the surrounding medium. It is notable that the thickness of those rod-string organelle screens is similar to the thickness of the multilayers in *Iridaea* [43].

The intra-cellular nanostructured organelles in red seaweeds have been recognized since the nineteenth century, although their functional purpose is still unclear. They could play a role in light management, as has been suggested for the brown alga *E. selaginoides* [20] and more specifically as a photoprotective mechanism, as in *Chondrus crispus* [22].

## 4. Phylogenetics, distribution and ecology

Little is known about the distribution of structural colour in seaweeds, with the article by Chandler *et al.* [19] being the only published work with a focus on the geographic distribution of structurally coloured brown seaweeds. Until now, no such work has been done for the rhodophytes. To build a baseline of data on the phenomenon for the present work, we investigated seaweed floras around the world [61–72] and published literature that mentioned the subject (e.g. [35,43,48]). We compiled a list of red algal species reported as having structural colour, which we used as the basis for investigating the algal herbarium at the Natural History Museum, London [73], for specimens of the given species. From these different sources, we extracted the data and analysed information on the geographic distribution and ecology of the species, as well as the metadata of the herbarium specimens (collection location, depth, mention of structural colour or iridescence etc.). A living database was created from this work and is added to as new information is found on the topic. This review is based on the list up to June 2025.

### 4.1. Phylogenetic distribution

The red seaweeds are thought to be at least 1.6 billion years old based on molecular clock analyses and fossil evidence [74]. This class is basal to the Florideophyceae which diverged between 817 million and 1 billion years ago and is the most taxon-rich class within the rhodophytes [75]. Within the red seaweeds so far, structural colour has only been reported in the Rhodymeniophycidae, a subclass within the Florideophyceae that emerged between 442 and 580 Ma [75].

**Figure 4.**
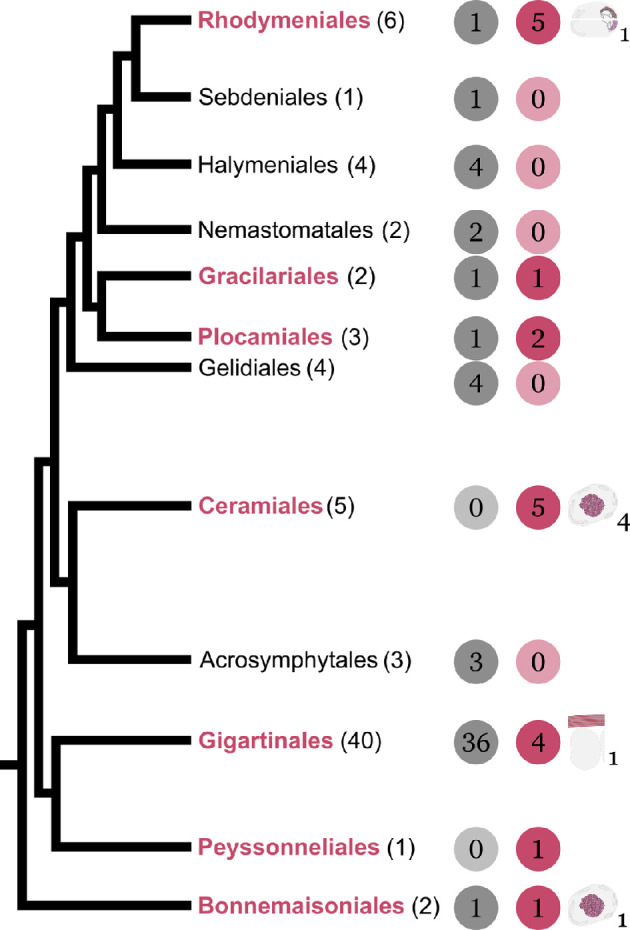
Simplified phylogenetic tree of the Rhodymeniophycidae orders containing structurally coloured species. In parentheses: total number of families in the order, based on current taxonomy [30]. In bold red font: groups containing species where structural colour has been reported. In grey circles: number of families where structural colour has not yet been reported. In coloured circles: number of families where structural colour has been reported. Where known, the type of nanostructure is indicated with a schematic (cf. figures 2 and 3) and the number of families in which the structure is reported is indicated in subscript. For further details at family, genera and species level in each order, see the supplementary material. Tree structure adapted from [75].

While most phycologists would generally name about five species of structurally coloured red algae (*Chondrus crispus* and *Chondria coerulescens* being the most renowned in western Europe, for example), results from the extensive herbarium study and literature research revealed that structural colour has in fact been reported in over 140 species belonging to 62 different genera, 18 families and 7 orders: the Rhodymeniales, Gracilariales, Plocamiales, Ceramiales, Gigartinales, Peyssonneliales and Bonnemaisoniales (cf. electronic supplementary material for details). A simplified phylogenetic tree of the Rhodymeniophycidae (figure 4) was adapted from Yang *et al.* [75], who constructed the phylogeny based on a combination of molecular markers (*rbc*L, *psa*A, *psb*A, EF2, SSU, LSU and cox1).

As shown in figure 4, the phylogenetic distribution of structurally coloured species is widespread but heterogeneous within the Rhodymeniophycidae, and not all families possess it. The phenomenon appears in groups that diverged at different times and is not found in all the descendant lineages of a common ancestor. While the oldest order (Bonnemaisoniales, *ca* 412 Ma) and the most recently evolved order (Rhodymeniales, *ca* 250 Ma) both contain structurally coloured species, the closest order to the Rhodymeniales to also include structural colour is the Gracilariales (*ca* 300 Ma), not the orders that diverged in the meantime (e.g. Sebdeniales, Halymeniales). The Gelidiales and Acrosymphytales have not been reported to possess structural colour, despite being paraphyletic with the Bonnemaisoniales, Peyssonneliales, Gigartinales, Ceramiales, Plocamiales and Gracilariales, all containing structurally coloured species.

In addition, as far as the available data show, structural colour is not always monophyletic within one order (cf. electronic supplementary material). For example, within the Gigartinales, structural colour is found in the Cystocloniaceae (e.g. *Hypnea caespitosa*), Gigartinaceae (e.g. *Chondracanthus cincinnus*), Rhizophyllidaceae (e.g. *Ochtodes akumalensis*) and Solieraceae (e.g. *Meristotheca procumbens*) but has not been reported in e.g. the Areschougiaceae or Dumontiaceae. On the other hand, within the more recent order Ceramiales, structurally coloured species have been reported from every family: Callithamaniaceae (e.g. *Aglaothmanion rigidulum*), Ceramiaceae (e.g. *Balliela crouanioides*), Delesseriaceae (e.g. *Acrosorium cilliolatum*), Rhodomelaceae (e.g. *Acanthophora pacifica*) and Wrangeliaceae (e.g. *Wrangelia argus*). In the Rhodymeniales, structural colour is reported in every family except one (Lomentariaceae).

One first hypothesis is that structural colour evolved in the Rhodymeniophycidae as the seaweeds moved to the shore following the rise of grazers during the post-Cambrian around 425 Ma [76]. The transition to the shore meant that the seaweeds became exposed to more intense sunlight and acquired a range of protection mechanisms, of which structural colour is a lesser-known type (e.g. protection of the genetic material in the gametes of *Chondrus crispus* [22]). This would mean that the structural colour would have been lost and retained in the successive divergences of the clade. However, closer inspection of the distribution of the types of nanostructures supports a different hypothesis.

While we lack comprehensive data on the type of nanostructures involved for each family, we observe that in the orders where we do have data, there is never evidence of nanostructures from two different categories found within the same order. One exception is found in the literature where, according to a description in Feldmann [34], *Ochtodes secundiramea* (order Gigartinales, family Rhizophyllidaceae) owes its purple iridescence to intra-cellular corpuscles in specialized cells. However, Feldmann seemed to equate these specialized cells to druse crystals (‘Drusenzellen’), which we consider to be different from the nanostructured organelles that cause structural colour, and, in the absence of a corresponding illustration in Feldmann’s description, we express doubts as to whether what he observed were truly the elements causing the purple iridescence visible on *O. secundiramea* thalli. In addition, considering the taxonomic complexity of the clade and the lack of recent systematic studies, the classification of the species is not certain. Due to these reservations, we have decided to rule out this exception until further study is possible. All other evidence from the literature points to an order-specific evolution of the type of structure involved: we find either intra-cellular or extra-cellular structures within one order, but not both (cf. electronic supplementary material).

In the Gigartinales, the extra-cellular multilayer structure is found in several genera of the Gigartinaceae family (e.g. *C. crispus* [18] and *Iridaea cordata* [38]). In the Ceramiales, intra-cellular nanostructured organelles are reported for the families Callithamniaceae, Ceramiaceae, Delesseriaceae and Rhodomelaceae [19,34,37,40,42,57]. In the Rhodymeniales, there is evidence for the intra-cellular nanostructured organelles (rod- or disc-shaped) in the Champiaceae family [34,36,40,43,48].

Although lack of evidence is not proof of absence, a possible hypothesis is that structural colour evolved more than once within the Rhodymeniophycidae, appearing in those orders through different mechanisms creating specific nanostructured architectures. It could conceivably be hypothesized that within the lineages that do contain structural colour, the type of nanostructure is probably lineage-specific and shared among all species with a common ancestor. To develop a full picture of the question, additional studies would need to look for gene signals across orders within the Rhodymeniophycidae.

To summarize, two evolutionary hypotheses can explain these findings: the structural colour trait could have appeared initially with the divergence of the Rhodymeniophycidae and then be lost in some subordinate taxa but retained in others. While this hypothesis would explain why the phenomenon is found in the more ancestral taxa (Bonnemaisoniales, Peyssonneliales, Gigartinales), it is difficult to support without evidence of structural colour in the Gelidiales, for example, and it does not explain why we find different types of nanostructures across the tree. The second hypothesis is that structural colour has evolved independently several times within the Rhodymeniophycidae, and that the taxa have independently created different nanostructured mechanisms for it in response to a variety of factors over geological time. Extensive molecular studies are needed to further understand the genetic evolution within the Florideophyceae.

### 4.2. Geographic distribution

With the data we compiled from the herbarium study and literature research, we assessed the distribution of structurally coloured red seaweeds around the world.

**Figure 5.**
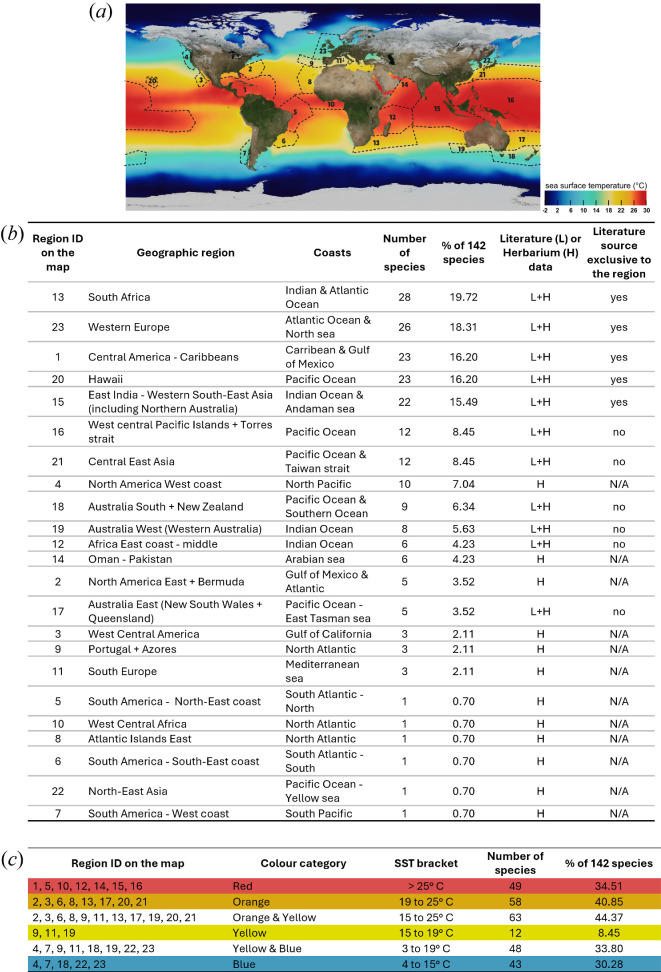
Geographic distribution of structurally coloured red seaweed species per regions of the world. (*a*) Map of the regions defined for the data analysis, in relation to sea surface temperature, using NASA’s global map of average sea surface temperature (SST) as a base (base map credits to: NASA/Goddard Space Flight Center Scientific Visualization Studio (https://svs.gsfc.nasa.gov/3652)). (*b*) Table of the species diversity for each region. (*c*) Table of the species diversity for each category of SST range. The colour code corresponds to the categories created based on the SST map. For (*b*) and (*c*), the first column corresponds to the numbered regions on map (*a*).

Structural colour in the red seaweeds has been reported from all main oceans (figure 5). The data presented were extracted from both the literature and herbarium study and categorized in four sea surface temperature (SST) groups based on NASA’s global map of average sea surface temperature [77] used as background: red, orange, yellow and blue (figure 5*c*). Excluding the ‘yellow’ category (SST between 15 and 19°C) which contains mostly herbarium data and a limited literature source that is not exclusive to the region, all three other categories contain data both from the herbarium and from literature exclusive to the region. Because of the nature of the data used, we excluded the ‘yellow’ category from the comparisons and found that the most records of structurally coloured species were from regions from the ‘red’ category (SST beyond 25°C) and the least number of species were reported in regions from the ‘blue’ category (between 4 and 15°C).

While we have focused on sea surface temperature in this study, several other oceanographic variables could be used to further explore the data. For example, water clarity and solar radiation are important factors which, in relation to the mechanisms and potential functions of structural colour, could reveal different kinds of patterns in the distribution of the phenomenon.

We found major clusters of structurally coloured species in South Africa (28 species), Western Europe (26 species), the Caribbean (23 species), Hawaii (23 species) and the eastern Indian Ocean (22 species). In the Caribbean and Hawaii, we found mostly species from the Rhodymeniales and Ceramiales. In Europe, South Africa and Australia, in addition to these two orders, we also found more species in the Gigartinales. These observed patterns must be interpreted with caution, as they could simply reflect where these orders are more diverse, inclusive of species with and without structural coloration.

### 4.3. Ecological distribution

**Figure 6.**
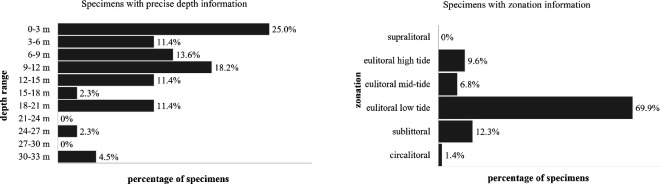
Distribution of the herbarium records per vertical zone of the littoral. The percentages correspond to a total of 44 specimens with precise depth information (left chart) and 73 specimens with zonation information (right chart).

By ‘ecological distribution’, we mean data relating to the environment in which the algal species are living. Specifically, here, we present data regarding the depth distribution of structurally coloured rhodophytes. Information on the ecological distribution of structural colour in seaweeds is scarce: within our data, as little as 23% of the 502 herbarium specimen records had information on depth, and only 8.8% had a precise depth figure while 14.5% only had information on the zonation (i.e. ‘eulittoral’, ‘sublittoral’ and so on). While the depth cannot be universally transcribed to a zonation, we observe that 36.4% of the records were above 6 m depth, which corresponds to the eulittoral (zone between the highest and lowest tides) in some regions, and 13.6% were between 6 and 9 m depth, which can correspond to the limit of the sublittoral. We also observe that 43.3% were reported between 9 and 21 m depth and 6.8% were deeper than 21 m depth (figure 6). The deepest record is from 33 m depth. Depth, along with turbidity, influences the amount of light reaching the organism, and thus the appearance and function of the structural colour [19].

These results on phylogenetic, geographical and ecological distribution are dependent on several factors such as the accuracy of taxonomic identification, given the high level of cryptic diversity in some taxa, or the geographic heterogeneity of sampling and recording efforts. The lack of data can be driven by several factors, mainly the problems of species identification and the time of recording [78]. It is important to note that the record of a species showing structural colour is not necessarily the affirmation that structural coloration has been observed in specimens from all localities for the said species. In addition, most collecting happens within the intertidal zone and on shallow dives (5–10 m [78]), which could influence the patterns observed in the ecological distribution. However, the size of the dataset used in this study gives us confidence that the figures extracted are representative of the bigger picture. Although knowledge of the specific structural colour mechanisms is too limited in each species to be able to segregate the data according to the types of structures (e.g. extra-cellular multilayer or intra-cellular nanostructured organelle), further research on structural colour in seaweeds should make it possible to gain a clearer understanding of the phylogenetic, geographical and ecological distribution of the different types of structures.

## 5. Biological significance and functional purpose

While biological significance of structural colour is well-studied in several animals, only a few studies exist for seaweeds [20–22,34,38]. Numerous authors who have observed structural colour in seaweeds have discussed hypotheses on its cause and role. Many authors have looked for a common cause across all species bearing structural colour, probably because, despite the variations, the phenomenon is quite similar across species in comparison with other forms of coloration. However, as Dixon [51] pointed out, the causes of structural colour may vary depending on the seaweed. The biological significance and functional purpose of structural colour in red seaweeds has never been experimentally researched until recently.

### 5.1. Photo-protection

In their studies on *Mazzaella flaccida*, *M. splendens* and *Iridaea cordata*, Gerwick & Lang [38] concluded that, for lack of further studies on the physiological effect of the absence or presence of the multilayered cuticle for those species, the structural colour seemed to be only a by-product of the secretion of a layered cuticle by the plant. If that is the case, the question remains, however, whether the by-product might confer enhanced survival abilities to the organism. Structural colour, whether initially a by-product or a purposeful evolution, is likely to serve a general role in light management at the cell level. Seaweeds are generally exposed to levels of light irradiance far above their physiological need. These high light levels cause damage to the photosynthetic apparatus, particularly in the intertidal zone. In response, seaweeds have evolved a range of mechanisms, such as non-photochemical quenching, to dissipate the energy at levels above what the photosystem can work with. However, this is not always efficient enough to protect against photo-damage. Oltmanns [79] suggested that structural colour is a protection mechanism against high light intensity and a way to discard the radiation harmful to the cell’s survival. This has been found to be the case for the hairy filaments on the Edelweiss flower [11], a different type of structural colour mechanism. While the efficacy of this role in seaweeds has been argued by subsequent authors [41,80], recent studies support it.

In fact, investigation of the light management in *Chondrus crispus* found that the multilayer cuticle helps the plant attenuate the more energetic wavelengths before they reach the photosystem, in order to prevent irreversible photo-damage in reproductive specimens. The multilayer cuticle influences the photosynthetic pathway used by the reaction centre, favouring the passage through the phycobilisomes, which can work as an intensity-dependent mechanism [22]. Considering that structural colour in *C. crispus* is only found in the gametophyte phase of the life history, this mechanism can be advantageous to protect the genetic material of the alga against UV-blue radiation which is expected in the shallow intertidal where this species grows [18,22] thus proving Oltmanns [79] hypothesis correct. This is also supported by personal observations on *Chondria coerulescens*, where the reproductive structures (the spermatangia, which produces the male gametes, and the carposorophyte, which develops post-fertilization on the female gametophyte) of the gametophyte phase display a vivid structural colour.

### 5.2. Photo-enhancement

In species of seaweeds living in zones of frequent exposure to high light, e.g. the intertidal species *C. coerulescens* and *Chondrus crispus*, the intra-cellular nanostructured organelles or the multilayers may help in shielding the seaweed against UV or in reflecting a subset of the light intensity in order to avoid damaging the photosystems. Alternatively, structural colour could act as a tool to manage low light intensities. Although these two hypotheses are often presented as mutually exclusive [34], this is not necessarily the case. In species adapted to low-light environments, e.g. *Aglaothamnion caudatum* or *Ochtodes secundiramea* (both collected by Feldmann [34] from the shallow subtidal in shady environments), the structures may serve to enhance the capture of the photosynthetically important wavelengths that reach their surface and direct it to the photosystems for a more efficient photosynthesis. This has been described in land plants such as *Selaginella uncinata* [81] and in shade-dwelling *Begonia* species [82], although no other study has supported these results and it has been refuted for *S. willdenowii* [2]. In shaded areas or in deeper waters where light is attenuated, structural colour mechanisms could act as a polarization mechanism to channel the photons for the better capture of light towards the photosystems within the cell [6,19]. Furthermore, a low-light-adapted organism which is less resilient to photodamage would also benefit from a mechanism allowing less light to penetrate inside the cells [2].

### 5.3. Protection against predation

Gerwick & Lang [38] also noted that the solidity of the cuticle of some Gigartinaceae could be an advantage in protecting the plant against grazing, especially for the more fertile specimens. This has not been further investigated in the reds, and from personal observations, we know that the structural colour apices in *C. crispus*, for example, are not particularly solid. Kawai & Motomura [83] note a correlation between the impact of herbivorous fishes on seaweeds and the presence or absence of structural colour in two closely related brown algal species from two different habitats, where the structural colour is caused by nanostructured organelles and thus not related to the cuticle. However, no studies have looked at the correlation between herbivore vision and the intensity of grazing which, if causal, could suggest that the colour itself had a role instead of being solely a consequence of the architectural structure of the cell or cuticle. This has been hypothesized in a more recent study on the red seaweed *Asparagopsis taxiformis*, where the structures causing the structural colour are also rich in noxious substances, and the resulting bluish and whitish colours would serve to camouflage the seaweed as less nutritious or toxic in the eyes of herbivores (aposematism) [59].

### 5.4. Visual attraction

Seaweeds are important habitat for species of invertebrates, some using them to camouflage themselves from predators. We can hypothesize that the structural colour could be exploited by these organisms for this purpose. In this case, the seaweed has not evolved in response to the animal but instead becomes a driver of evolution in other organisms.

Lavaut *et al.* [84] demonstrated that the fertilization process of the red alga *Gracilaria gracilis* (Gracilariales, Gracilariaceae) is increased by enhanced interaction with an isopod via the dissemination of the gametes on the seaweed’s thallus. *Gracilaria gracilis* is not structurally coloured; however, these findings let us imagine that structural colour could also act as an attraction mechanism for animals with adequate vision, who would act as mediators for the fertilization of the seaweed, in the same way it has been described for some terrestrial plants. The iridescence caused by structural colour in some flowers has been thought to be used as pollination cues by pollinating insects [14,17,85], although this is not unanimously accepted [86]. The difficulty of assessing how animals perceive colours brings an additional challenge to understanding the role of structural colour in plant–animal interactions [16]. Further research with more focus on such interactions would be needed to clarify whether these hypotheses are worth considering.

## 6. Conclusion and future directions

This review set out to lay out and reframe the current extent of knowledge on structural colour in red seaweeds. The main finding of this investigation is that structural colour in red seaweeds is more diverse and widespread than had been reported, both in terms of mechanisms and distribution. The study has identified two main categories of nanostructures, which we call extra-cellular multilayers and intra-cellular nanostructured organelles, and sub-types within that second category. One of the more significant findings to emerge from this investigation is that these nanostructure types appear to be lineage-specific. Another major finding from the combined literature and algal herbarium study is that structural colour is in more red algal orders than had been recorded by sporadic reporting, with higher phylogenetic and geographic diversity. This review has also put into perspective the main hypotheses currently existing on the biological functions of structural colour for red seaweeds, arguing that there is no single explanation to the phenomenon and that structural colour has different causes and roles across species.

This is a fruitful area for further work. Taxon-wide genomics and transcriptomics studies are needed to explore gene signals across the phylogenetic tree and shed light on the evolutionary history of the phenomenon. A greater focus on the nanostructured organelles, in particular, could produce interesting findings that would greatly deepen our understanding. Chemical analyses should be carried out to establish the composition of the nanostructured organelles in each genus. Extensive electron microscopy work is required to determine how they are generated and how they develop throughout the life history of the seaweeds. Further experimental investigations combining cultures and microscopy analyses monitoring their response to changing light conditions would help elucidate the precise mechanism by which they function. With further research in those directions, it should become possible to replicate these mechanisms in the future and apply these findings to the design of biomimetic novel technologies for light management. Moreover, understanding the evolutionary dynamics involved would enlighten us on the future distribution of seaweeds in relation to climate change.

As we focus on the phenomenon, we notice species with previously unreported structural colour in almost every region we undertake fieldwork in. Continued efforts are needed to report and record structural colour in seaweeds in order to generate a more comprehensive picture of the phenomenon.

## Data Availability

The data used in section 4 of this paper is available on Zenodo at doi: [87]. Supplementary material is available online [88].
